# Nomenclature in Abdominal Wall Hernias: Is It Time for Consensus?

**DOI:** 10.1007/s00268-017-4037-0

**Published:** 2017-05-01

**Authors:** Samuel G. Parker, Christopher P. J. Wood, David L. Sanders, Alastair C. J. Windsor

**Affiliations:** 1 Department of General and Colorectal Surgery, University College London Hospital, 235 Euston Road, London, NW1 2BU UK; 2 Department of General and Upper GI Surgery, North Devon District Hospital, Raleigh Park, Barnstaple, Devon EX31 4JB UK

## Abstract

Abdominal wall reconstruction is a rapidly evolving area of surgical interest. Due to the increase in prevalence and size of ventral hernias and the high recurrence rates, the academic community has become motivated to find the best reconstruction techniques. Whilst interrogating the abdominal wall reconstruction literature, we discovered an inconsistency in hernia nomenclature that must be addressed. The terms used to describe the anatomical planes of mesh implantation ‘inlay’, ‘sublay’ and ‘underlay’ are misinterpreted throughout. We describe the misinterpretation of these terms and give evidence of where it exists in the literature. We give three critical arguments of why these misinterpretations hinder advances in abdominal wall reconstruction research. The correct definitions of the anatomical planes, and their respective terms, are described and illustrated. Clearly defined nomenclature is required as academic surgeons strive to improve abdominal wall reconstruction outcomes and lower complication rates.

## Introduction

The repair of complex ventral hernias (CVHs) is a rapidly evolving area of surgical interest. Complex hernias are becoming both increasingly prevalent [1] and challenging [2], with a consequent need for the academic hernia community to produce robust research to guide best practice. It is clear when reviewing the CVH repair literature that the nomenclature is used with significant variability and often incorrectly. For example, the recto-rectus plane is often referred to as the ‘inlay’ [3, 4], ‘sublay’ [5–8] or ‘underlay’ [9] plane. The pre-peritoneal layer is often also referred to by all three terms; ‘inlay’ [10], ‘sublay’ [11] and ‘underlay’ [12]. And finally, the intra-abdominal plane is often referred to as ‘sublay’ [13] or ‘underlay’ [14, 15].

Attempts to produce evidence to guide the best surgical management of these CVH repairs is already challenging, given the considerable pre-operative and peri-operative variables in these patients. Therefore, it is imperative for surgeons and researchers to use standardised correct nomenclature to prevent misinterpretation, to reduce data heterogeneity and allow for accurate study comparison.

## Evidence of inconstancy in the literature: are we using the same language?

**Fig. 1:**
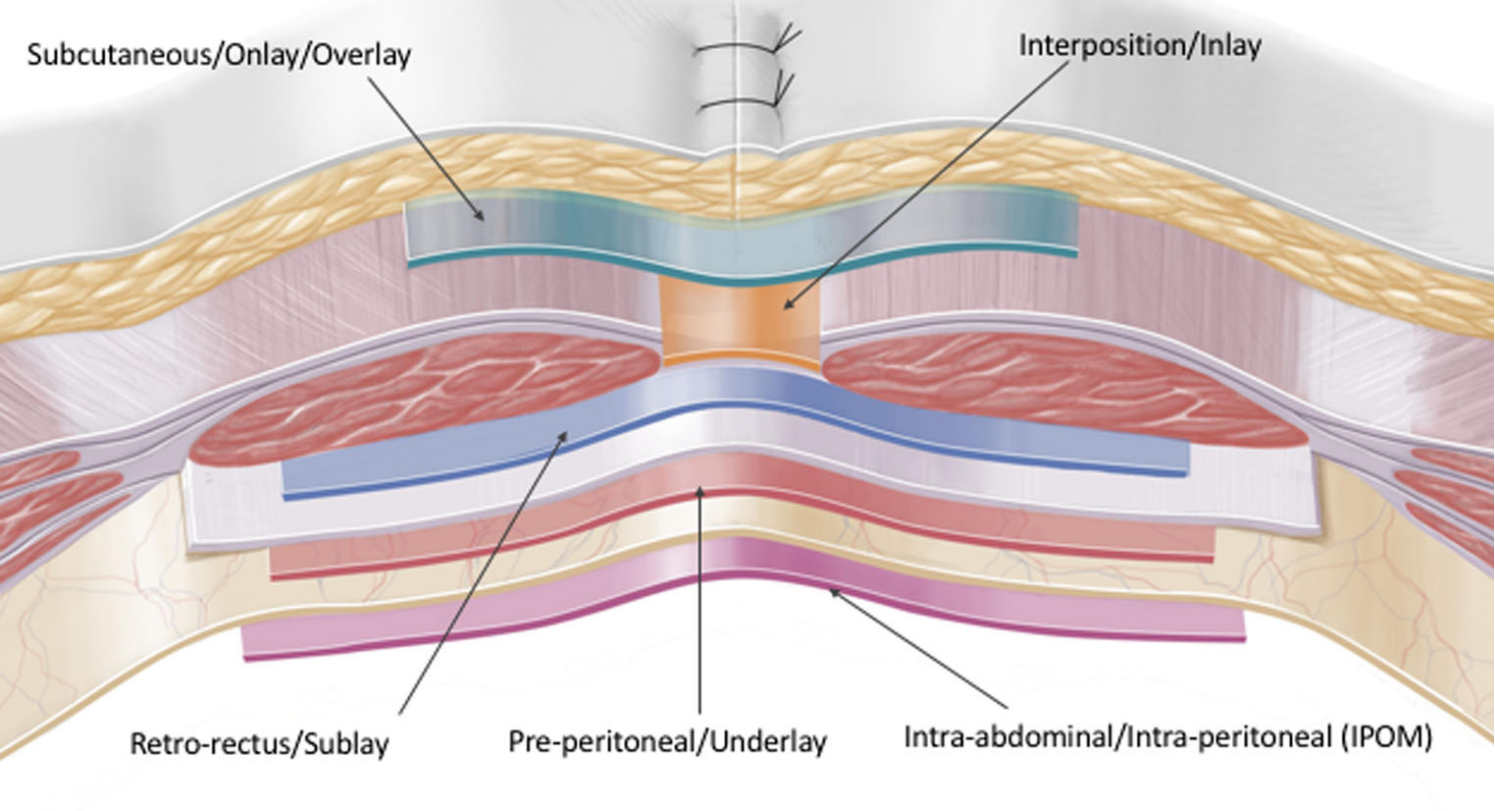
[31] Illustration clearly showing the planes of the anterior abdominal wall

As discussed, review of the CVH literature demonstrates multiple examples of inconsistent nomenclature usage. These inconsistencies become of increased concern when these terms are used for intra-operative variable analysis in systematic reviews [15, 16] and meta-analysis [17, 18], which have a greater potential to influence wider clinical practice. In a much-cited Cochrane review [16], 5 RCTs [19–23] are meta-analysed to compare local wound complication rates of open ‘sublay’ repairs versus laparoscopic repairs. Critical analysis shows that in two [20, 23] of the RCTs the mesh was in fact inserted in the ‘underlay’ plane, i.e. pre-peritoneal and not retro-rectus (see Table 1; Fig. 1). As a result, this review pools RCTs with open sublay and underlay repairs into a larger ‘sublay’ group and compares their local wound complication rates to laparoscopic repair. The evidence must therefore be interpreted with some caution as the premise is misguided and wrongly assumes that all five trials used an open technique with the mesh in the ‘sublay’ rectro-rectus plane.

**Table 1:** Defining the planes of the anterior abdominal wall

Detailed anatomical description	Abbreviated anatomical terms	Ventral hernia nomenclature/colloquial terminology
Mesh is laid on top of the external oblique over the defect	Subcutaneous/onlay/overlay	Onlay/overlay
Mesh is the same size as the hernia defect and the edges are sutured the hernia neck	Inlay/interposition (always bridging)	Inlay (always bridging)
Posterior to the rectus muscles and anterior to the posterior rectus sheath^a^	Retro-rectus	Sublay
Anterior to the peritoneum and posterior the rectus sheath^b^	Pre-peritoneal	Underlay
Mesh is inserted into the Abdominal compartment and laid on the anterior abdominal wall deep to the peritoneum. Often bridging especially in laparoscopic surgery	Intra-abdominal/intra-peritoneal onlay mesh (IPOM)	Intra-peritoneal/(IPOM)

^a^Below the arcuate line this layer is between the rectus abdominis muscles and the transversalis fascia. After TAR this layer extends laterally between the transversalis fascia (posteriorly) and the transversus abdominis muscle (anteriorly)

^b^Below the arcuate line the peritoneum is posterior and the transversalis fascia is anterior. Lateral to the posterior rectus sheath this layer is between the peritoneum (posteriorly) and the transversalis fascia (anteriorly)

Further interrogation of the literature reveals other examples of error stemming from nomenclature inconsistencies. A meta-analysis comparing onlay and sublay hernia repairs includes one study [24] that does not use the sublay plane at all, being reported as ‘underlay’ in the original paper. For a genuine sublay versus onlay meta-analysis, this RCT should have been omitted. Many other examples of the inconsistent use of the nomenclature exist, but these examples demonstrate our point that mixing up the mesh planes in meta-analysis increases heterogeneity and leads to misleading outcomes.

## Why is the nomenclature important?

Precise nomenclature describing the abdominal wall planes is important for three reasons. Firstly, the position of the mesh affects the mechanisms of hernia recurrence. For example, intra-abdominal and pre-peritoneal meshes are placed posterior to the transversalis fascia. Consequently, there is a potential for failure at the mesh-fascia interface and for hernia recurrence via either ‘lateral detachment of the mesh’ or ‘inadequate mesh fixation’. These two mechanisms of recurrence also exist for implanted inlay mesh but not for mesh placed in the sublay and onlay planes [25]. Each mesh plane has its own individual set of mechanisms of recurrence, and therefore each plane should be considered independently and not grouped together when analysed.

Secondly, the literature already reports that all post hernia repair complication rates (not just recurrence) are influenced by where the mesh is placed [15, 17]. For example, intra-abdominal mesh exposed to abdominal viscera increases the risk of adhesions, bowel obstruction and fistula formation [26, 27]; onlay mesh placement is associated with higher wound infection rates [15, 17] and hernia recurrence rates are reduced with the mesh in the retro-rectus (sublay) position [17, 26]. As evidence to guide clinical practice emerges, precise and consistent nomenclature is essential to interpret complication rates relative to the respective anatomical plane.

Lastly, the biomechanics of the abdominal wall are complex. The multiple fascial (collagen) and muscular (muscle fibres) layers each have their own elasticity, tensile strength and anisotropic configuration [28, 29]. Meshes are clearly not as dynamic and shear forces occur at the points of mesh fixation. This causes tearing of collagen and muscle fibres with subsequent defects and hernia recurrence [28, 30]. Research is therefore required to reduce these shear forces and maximise the physiological function of the abdominal wall post repair. Physiological meshes need to be synthesised and placed in specific anatomical planes. This will allow us to identify optimal mesh fixation techniques and to study the forces that occur between the mesh and the adjacent in vitro abdominal wall layers.

## Correct nomenclature: unified approach based on anatomical accuracy

We recommend that consistent nomenclature is used, based on a detailed appreciation of the abdominal wall anatomy. The correct anatomical description of the mesh planes in the abdominal wall is shown in Table 1 and Fig. 1 [31]. This has been described in the literature [32, 33] but has never been laid out in such detail. This anatomical description is shown with the correct abbreviated anatomical terms. The correct nomenclature or ‘colloquial’ terminology is also defined [32, 33].

## Conclusion

The repair of CVH is a challenging area in both surgical practice and surgical research. Inconsistencies in the understanding of the nomenclature and the anatomy are leading to flaws in the data. This has the potential to be misleading and generate spurious evidence. We recommend that a consistent nomenclature based on an appreciation of the anatomy is used. This will aim to ensure that not only is the clinical management consistent, but allows for a transparent and unified evidence base for these complex surgical cases.
